# Severe type-B lactic acidosis in a patient with bilateral renal Burkitt’s lymphoma 

**DOI:** 10.5414/CNCS110123

**Published:** 2021-04-26

**Authors:** Juan D. Salcedo Betancourt, Oscar A. Garcia Valencia, Victor G. Becerra-Gonzales, Karla G. Carias Martinez, Jennifer Chapman, Natalia Yanchenko, Marco A. Ladino

**Affiliations:** 1 Department of Medicine, Nephrology Section, University of Miami Miller School of Medicine/Miami VAMC, and; 2 Department of Pathology and Laboratory Medicine, University of Miami, Jackson Memorial Hospital, Miami, FL, USA

**Keywords:** lactic acidosis, Burkitt’s lymphoma, non-Hodgkin lymphoma, kidney neoplasms

## Abstract

Introduction: Lactic acidosis (LA) can be categorized as type A, which occurs in the presence of tissue hypoxia, or type B, occurring in the absence of tissue hypoxia. Hematologic malignancies are an uncommon cause of type B LA. Case presentation: A 63-year-old man, HIV-negative, with a history of diabetes mellitus, hypothyroidism, and non‐alcoholic fatty liver disease (NAFLD), presented to the ED complaining of acute-on-chronic lumbar pain, and was found to have high serum anion gap (AG) LA. The rest of chemistry and infectious workup was within normal limits. Despite bicarbonate therapy and fluid resuscitation, the patient remained with persistent AG metabolic acidosis and increasing lactic acid up to 14.5 mmol/L. An abdominal computerized tomography (CT) revealed multiple bilateral enhancing lesions in the kidneys, as well as gastric wall thickening. Upper gastrointestinal endoscopy with biopsy showed a high-grade Burkitt’s lymphoma. Further staging showed bone marrow involvement and extensive abdominal adenopathy. After two cycles of inpatient chemotherapy with dose-adjusted EPOCH-R (etoposide, prednisone, vincristine, cyclophosphamide, doxorubicin and rituximab), the patient developed multifocal pneumonia complicated by respiratory failure. Following a prolonged ICU stay, after discussion with the family members, a decision of withdrawal of life-sustaining therapy was reached. Conclusion: Persistent LA, without identifiable causes of tissue hypoxia, should prompt clinicians to suspect non-hypoxic etiologies, including occult high-grade malignancies. Hematological malignancies constitute an extremely rare cause of type-B LA, carrying a poor prognosis.

## Introduction 

Under anaerobic conditions, lactic acid is produced from pyruvate at the end of the glycolytic pathway. It is primarily produced from skeletal muscle (25%), skin (25%), brain (20%), erythrocytes (20%), and the intestine (10%), and is metabolized by the liver (60%) and the kidney (30%), where it is a substrate for gluconeogenesis [1, 2]. 

Basal lactate production is ~ 0.8 mmol/kg/hour, with a normal serum lactate concentration of < 2 mmol/L [3, 4]. Intermediate lactic acidosis (LA) is defined as levels > 2 mmol/L, and severe LA (≥ 4 mmol/L) is associated with an increased mortality risk, independently of organ failure and shock [5]. LA usually occurs with an elevated serum anion gap above 12 mEq/L, due to secondary loss of bicarbonate from buffering [1]. 

LA can occur from either increased production and/or decreased clearance, and can be categorized as either type A, which occurs in the presence of tissue hypoxia, or type B, occurring in the absence of tissue hypoxia. Type A LA is associated with conditions such as: systemic hypoperfusion (shock), local hypoperfusion (limb or mesenteric ischemia), severe hypoxemia, severe anemia, carbon monoxide poisoning, or increased glycolysis (seizures, exercise). On the other hand, type B LA occurs when increased glucose metabolism exceeds the oxidation capacity of the mitochondria, thereby increasing lactate production, and has been associated with conditions such as: underlying diseases (liver disease, human immunodeficiency virus, malignancies, thiamine deficiency, diabetic ketoacidosis, pheochromocytoma), medications/toxins (β2-agonists, toxic alcohols, metformin, salicylates, acetaminophen, cyanide, propofol), and inborn errors of metabolism (mitochondrial myopathies, pyruvate dehydrogenase deficiency, among others) [1, 2, 3]. Hematologic malignancies are an uncommon cause of type B LA, usually carrying a very poor prognosis [6]. We describe a case of persistent LA that led to a discovery of underlying Burkitt’s lymphoma. 

## Case report 

**Figure 1:**
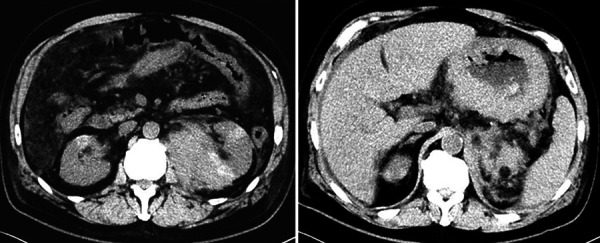
Abdominal CT scan with contrast. Findings of bilateral enhancing lesions in the kidneys. Multiple rounded hyperdense lesions throughout the renal parenchyma bilaterally, more pronounced in the left kidney. Left perinephric hematoma 8.8 × 5.2 cm. Axial view (left). Diffuse thickening of the stomach wall. No evidence of liver lesions. Axial view (right).

**Figure 2:**
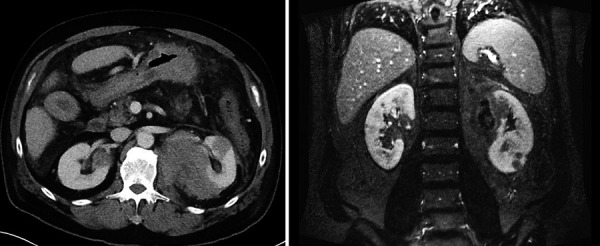
Abdominal MRI. Multiple rounded hypo enhancing lesions noted throughout the renal parenchyma bilaterally. Axial view (left). Coronal view (right).

**Figure 3:**
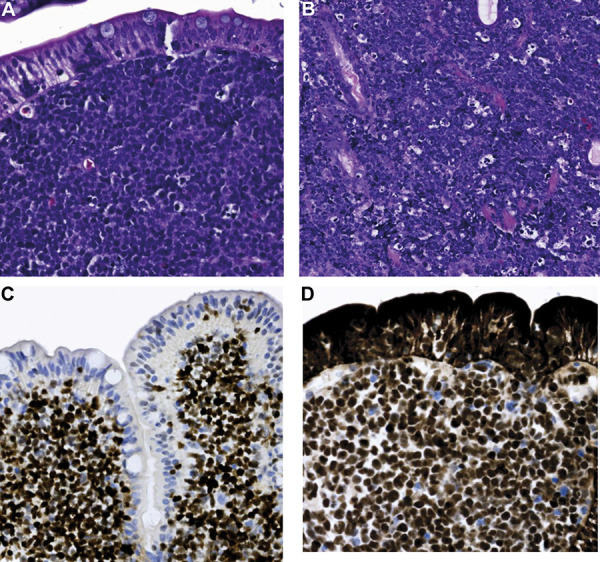
Microscopic appearance of the high-grade Burkitt’s lymphoma. Stomach. Gastric mucosa with a dense lymphoid infiltrate in the lamina propria consisting of intermediate size monotonous lymphoma cells with round nuclei, fine chromatin, and multiple nucleoli. Mitoses are conspicuous. Multiple apoptotic bodies impart a starry sky pattern to the lesion (A, B: hematoxylin & eosin). Immunohistochemistry was positive for CD20, CD10, BCL6 (C), c-MYC (D), and MUM1, and are negative for CD5, BCL2, CD30, and terminal deoxynucleotidyl transferase (TdT). Fluorescence in situ hybridization (FISH) was 73% positive for MYC-IGH fusion and negative for IGH-BCL2 and BCL6 rearrangements.

A 63-year-old HIV-negative man, with a history of well controlled diabetes mellitus, hypothyroidism, and Non‐alcoholic fatty liver disease (NAFLD), presented to the ED complaining of acute-on-chronic lumbar pain. His home medications included insulin, atorvastatin, and levothyroxine. Upon arrival, patient was afebrile, with a blood pressure (BP) of 86/52 mmHg with orthostatic changes, rest of vital signs within normal limits. Patient was found somnolent, with tenderness on the left upper quadrant, without rebound. Rest of physical exam was within normal limits. Laboratory results showed a hemoglobin 9.4 g/dL (14 – 18 g/dL), leukocytes 17 × 10^3^ leukocytes/µL (4 – 11 × 10^3^/µL), serum bicarbonate 15 mmol/L (22 – 29 mmol/L), serum lactate 5.0 mmol/L (0.5 – 1.8 mmol/L) with anion gap (AG) LA of 28 (6 – 12), arterial blood gases pH 7.26 (7.38 – 7.44), paO_2_ 83.4 mmHg (75 – 100 mmHg), paCO_2_ 24.5 mmHg (38 – 42 mmHg), HCO_3_ 10.8 mmol/L (23 – 26 mmol/L). Lactate dehydrogenase (LDH) was 1,041 U/L (80 – 225 U/L). The rest of chemistry and infectious workup was within normal limits. Of note, 2 months prior to presentation, patient was incidentally found to have a left perinephric hematoma after he had an abdominal computer tomography (CT) for NAFLD workup. Upon admission, a repeat abdominal CT scan showed interval increase of the perinephric hematoma as well as gastric wall thickening (Figure 1). Urology recommended no surgical intervention. He was resuscitated with isotonic intravenous fluids, and was admitted to the intensive care unit (ICU), where he continued supportive care. Two days after admission, despite bicarbonate therapy and fluid resuscitation with complete resolution of shock, the patient remained with persistent AG metabolic acidosis and increasing lactic acid up to 14.5 mmol/L. A subsequent abdominal magnetic resonance imaging (MRI) showed multiple rounded hypoenhancing lesions noted throughout the renal parenchyma bilaterally (Figure 2). A kidney biopsy was planned but aborted since the patient became acutely anemic (hemoglobin 5.6 g/dL) after an episode of melena. Upper gastrointestinal endoscopy with biopsy showed a high-grade Burkitt’s lymphoma (Figure 3). Further staging showed bone marrow involvement and extensive abdominal adenopathy. After two cycles of inpatient chemotherapy with dose-adjusted EPOCH-R (etoposide, prednisone, vincristine, cyclophosphamide, doxorubicin, and rituximab), the patient developed multifocal pneumonia complicated by respiratory failure. Following a prolonged ICU stay, after discussion with the family members, a decision of withdrawal of life-sustaining therapy was reached. 

## Discussion 

Malignancy-related LA is defined as a plasma level > 4 mmol/L in the setting of a tumor, even in the absence of overt acidemia [7]. LA has been associated with both solid (15%) and hematological malignancies (85%), of which non-Hodgkin’s lymphoma (NHL) accounts for 50% of cases [8, 9]. However, type B LA in the setting of NHL is an uncommon and devastating complication, carrying a very poor prognosis with a mortality rate of up to 92% [6, 9, 10]. 

Burkitt’s lymphoma is an uncommon and highly aggressive B-cell lymphoma accounting for < 1% of adult NHL [11]. It most commonly affects the ileocecal area; however, it may also involve extra nodal sites (such as bone marrow, ovaries, kidneys, and breasts) [12]. Renal involvement is usually asymptomatic [13], and requires a high degree of suspicion to prevent early complications such as multisystem organ dysfunction [14]. Only a few cases of severe LA in patients with Burkitt’s lymphoma have been previously reported in the literature [6, 15, 16, 17, 18, 19, 20]. 

It is proposed that an acidic microenvironment is critical for tumor progression, since extracellular acidity induces genome instability, tumorigenesis, angiogenesis, and metastasis. Also, lactate and acidosis have shown to inhibit anti-tumor immune surveillance [21, 22]. Suggested mechanisms for malignancy-related LA include: the Warburg effect (increased glycolytic activity of malignant cells), tumor tissue hypoxia leading to anaerobic glycolysis, and decreased lactate clearance in the setting of liver metastases [1, 6, 23]. At the molecular level, it has been shown that c-MYC induces overexpression of glycolytic enzymes, glucose transporters, and lactate dehydrogenase [24]. In our case, it is also possible that the lymphomatous kidney infiltration may have contributed to decreased lactate renal clearance, as it has been previously described [11]. 

## Conclusion 

LA is commonly used as a marker for tissue hypoxia in the critically ill patient (type A). However, it may also occur in the absence of tissue hypoxia (type B), when increased glucose metabolism exceed the oxidation capacity of the mitochondria, thereby increasing lactate production. Hematologic malignancies are an unusual etiology of type B LA, usually carrying a devastating prognosis. Persistent LA, without identifiable causes of tissue hypoxia, should prompt clinicians to suspect non-hypoxic etiologies, including occult high-grade malignancies. Treatment should focus on identifying and correcting the underlying etiology. 

## Funding 

The authors received no funding for this project. 

## Conflict of interest 

The authors have no conflicts of interest to disclose.
